# Determinants of Awareness of High School and Preparatory Students About HIV/AIDS: The Case of Sayint Preparatory and General Secondary School, South Wollo, Ethiopia

**DOI:** 10.1002/puh2.70357

**Published:** 2026-09-10

**Authors:** Tilahun Adugna, Solomon Gezahegn, Fitsum Tigu, Mujib Abdulkadir, Asnake Desalegn

**Affiliations:** ^1^ Department of Zoological Sciences College of Natural and Computational Sciences Addis Ababa University Addis Ababa Ethiopia; ^2^ Department of Medical Laboratory School of Medicine Goba Referral Hospital Madda Walabu University Robe Oromia Ethiopia; ^3^ Department of Microbial Sciences and Genetics College of Natural and Computational Sciences Addis Ababa University Addis Ababa Ethiopia

**Keywords:** attitude to health, health knowledge, human immunodeficiency virus (HIV) infection, practice

## Abstract

**Introduction:**

The human immunodeficiency virus (HIV) continued to be the major threat to the world. The Ethiopian National Plan, as well as the UNAIDS target for the year 2021–2025, is to significantly reduce the transmission, enhance the prevention and control strategies to reduce deaths from the disease.

**Objective:**

This study aimed to investigate the determinants of knowledge, attitude, and practice (KAP) towards HIV/AIDS among Sayint Preparatory and General Secondary School students.

**Methods:**

An institution‐based cross‐sectional study was conducted from August to September 2023. Purposive sampling was used to select the site, and simple random sampling was used to recruit study participants.

**Results:**

The majority of the respondents had good knowledge (87.3%), positive attitude (88.9%), and good practice (88.6%). From the multivariate analysis, being from an urban area (AOR = 6.52, CI: 2.22–19.12), Grade 10 (AOR, 2.70; CI: 1.15–6.36), and Grade 11 (AOR, 3.69; CI: 1.47–9.30) and (AOR, 2.41–30.27) were factors associated with the knowledge about HIV/AIDS. Being married (AOR, 3.98, CI: 1.10–14.56) and obtaining information from family members (AOR, 0.30; CI: 0.11–0.80) were variables significantly associated with attitude towards HIV/AIDS.

**Conclusion:**

The KAP of the respondents towards HIV/AIDS was high, but misconceptions were also noted. Extensive sex education programs in schools should be devised to solve the risks associated with HIV/AIDS.

## Graphical abstract

• The study participants have good knowledge, attitude, and practices.

• But the level of misconception among minorities could potentially lead to contracting the disease.

• Some level of stigma towards people living with HIV/AIDS was also noticed among some participants.

• Student‐tailored advocacy or school training is very important.

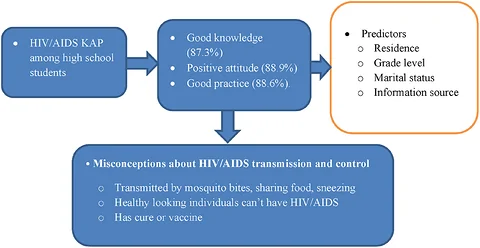

## 1 Introduction

The human immunodeficiency virus (HIV) has been a major health issue worldwide since the disease was first recognized [1]. According to WHO regional data in the year 2022, there were 39 million people living with HIV, 1.3 million acquiring HIV, and 630,000 died of HIV‐related causes. Among the people living with HIV, the majority (25 million) were from Africa. From the African regions, more than 20 million were from Eastern and Southern Africa [2]. In Ethiopia, the prevalence of HIV/AIDS differs from one region to the other. HIV prevalence was the highest in *Gambella* town among adults of 15–49 and adolescent and young people from 15 to 24 years of age. Regarding the regional distribution of HIV/AIDS, the highest population of people living with HIV/AIDS and new infections was reported from Amhara region followed by Oromia (NSP, 2021–2025).

The UNAIDS treatment target for 2021–2025 is represented as 95‐95‐95, signifying that 95% of people living with HIV know their status, 95% of those who know their status access treatment, and 95% of those on treatment have suppressed viral loads in the years 2021–2025 [2]. Similarly, Ethiopia has an AIDS‐free National vision for the year 2021–2025 of reducing HIV infection and HIV‐related deaths to less than 1 per 10,000 populations (NSP, 2021–2025).

To achieve the strategic objectives of reducing HIV infection and mortality by the year 2025, assessing the level of knowledge, attitude, and practice (KAP) of the community seems indispensable. Reports indicate that studies on the KAPs about HIV/AIDS are important determinants of the quality care provided to HIV/AIDS patients, planning the management and prevention of HIV [3, 4, 5]. Despite being among the high‐burden countries with respect to HIV/AIDS, community‐based research on KAPs is limited.

A study in the Amhara region, Gondar city, Ethiopia, reported underutilization of HIV testing and counseling services [6]. There was a low level of HIV/AIDS knowledge, a high level of misconception about HIV/AIDS, and stigma against people living with HIV/AIDS [7]. High awareness about HIV/AIDS but risky sexual behaviors like having multiple sexual partners and low levels of condom use were also reported [8]. In more recent surveillance data from 2019 to 2021, high seroprevalence of HIV was reported among urban centers [9]. Therefore, the KAP studies help to identify knowledge gaps about HIV/AIDS as poor knowledge could contribute to the spread of HIV [10]. Lack of information or misinformation makes adolescents highly vulnerable to HIV/AIDS, and help in school‐based interventions to solve negative and discriminatory attitudes [11]. Besides, it helps to tailor HIV prevention strategies to specific age groups and cultural contexts [12]. To the best of our knowledge, there has not been any published document on the determinants of KAP in the study setting. Therefore, the study aimed to assess the determinants of KAP of high school students towards HIV/AIDS.

## 2 Methodology

### 2.1 Description of the Study Area

**FIGURE 1:**
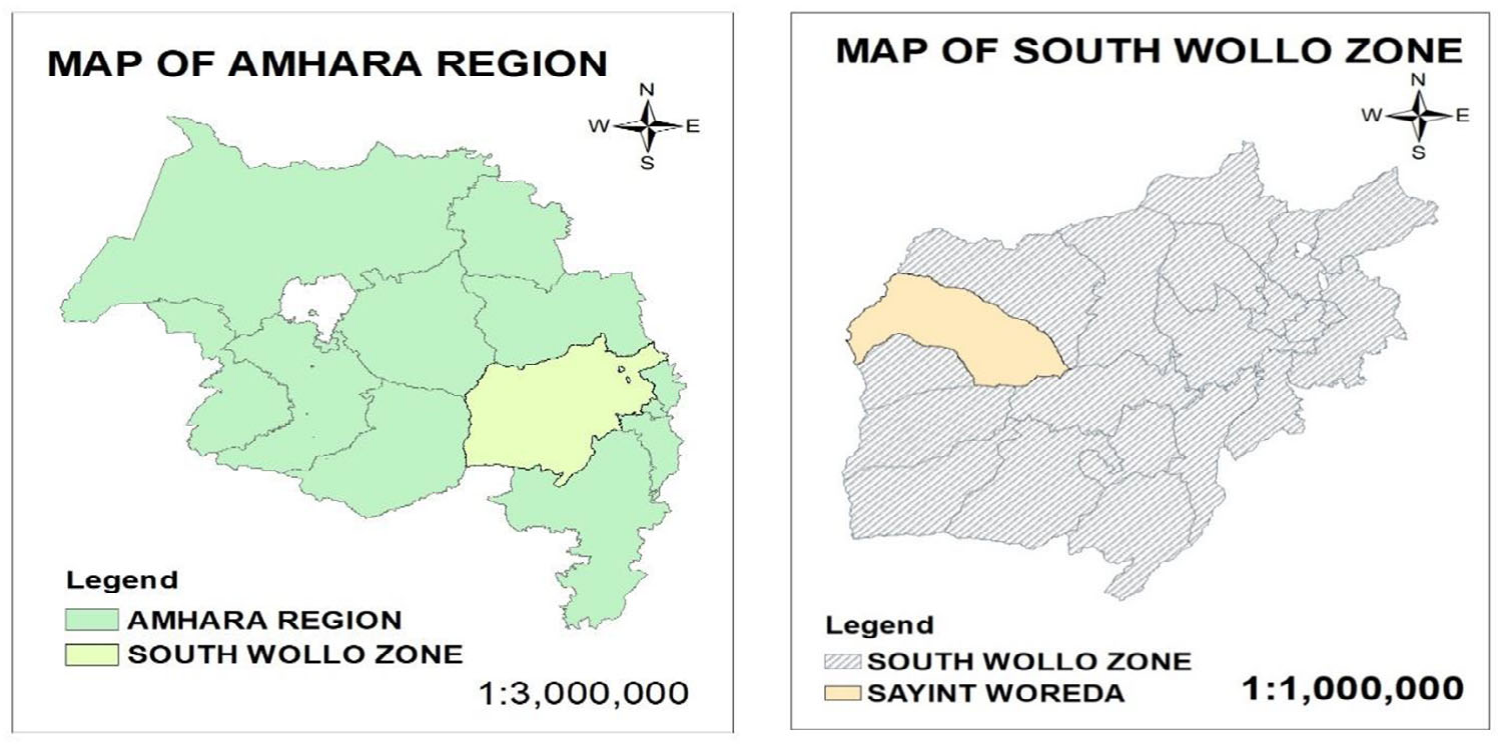
Map of the study area.

Amhara Sayint *Woreda* (*disrtict*) is one of the districts of South Wollo Zone, Amhara Region, Ethiopia. *Adjibar* is the main town of the district (Figure 1). It is located at about 187 km distance from Dessie city in the southwest direction and at about 590 km north of Addis Ababa, the capital city of the country (Amhara Sayint *Woreda* Communication Office, 2023). Amhara Sayint Adjibar General Secondary and Preparatory School was established in 1992 G.C. The school provides education services from Grade 9 up to Grade 12 and has 3200 students, of which 1750 are female and 1450 are male students.

### 2.2 Study Design

The study design was an institution‐based cross‐sectional study conducted among high school and preparatory school students from Sayint Secondary and Preparatory School.

### 2.3 Study Population and Sampling Procedures

The study population constituted Grades 9–12 students selected from Sayint Preparatory and General Secondary School during the academic year 2023. The school was purposively selected because of the absence of KAP study on HIV/AIDS and being the only oldest preparatory and high school in the area. After the students’ lists were obtained from the schools, they were stratified by grade levels, and proportionate allocation was done for each grade. Finally, from each stratum (grade) study participants were recruited through simple random sampling technique. The sample size was determined using single population proportion formula, and sample size adjustment was made as the total population was less than 10,000. Finally, 10% nonresponse rate was considered, and a total of 377 study participants were recruited as indicated below:

$$n_{i}=\frac{{\left(Z\alpha /2\right)}^{2}Xp\left(1-p\right)}{d^{2}\;}=\frac{\;\left(1.96\right).5\left(1-0.5\right)}{{\left(0.05\right)}^{2}}=384$$

where (Z α/2) = critical value for normal distribution at 95% confidence interval, 1.96; *p* is the population proportion, 0.5; *d* is the margins of error, 0.05; and *n_i_* is the sample size before adjustment, 384.

Accordingly, the sample size before adjustment was 384. As the source population was less than 10,000, a sample size adjustment was made using the following formula, and with 10% nonresponse rate of 34, the total population size was 377.

$$\mathrm{NF}=\frac{n_{i}}{1+\frac{n_{i}}{N}}=\frac{384}{1+\frac{384}{3200}}=343$$

The number of samples from each grade level was determined using the proportional allocation formula below, where *n*_h_ represents the number of samples drawn from grade h, *N*_h_ is the population size of grade h, *N* is the total population size across all grades, and *n* is the overall sample size. The number of students selected from each grade was proportional to the total number of students in the specific class. The population size per grade was 968, 858, 781, and 593 for 9th, 10th, 11th, and 12th grades, respectively.

$$n_{h}=\left(N_{h}/N\right)\times n$$

### 2.4 Data Collection

Data were collected from August 31, 2023, to September 29, 2023, using pretested self‐administered structured questionnaires comprised of sociodemographic variables, knowledge about HIV/AIDS, attitude towards HIV/AIDS, and HIV/AIDS‐related practices. The pretesting was conducted on 5% of the total sample size among students with similar backgrounds but not included in the final study. The questionnaire was initially prepared in the English language and translated to the local language (Amharic) by a bilingual expert and finally translated back to English by another expert fluent in both languages. The internal consistency of the questionnaire was assessed using Cronbach's alpha. The reliability coefficients for each category were as follows: knowledge (*α* = 0.88), attitude (*α* = 0.87), and practice (*α* = 0.76).

### 2.5 Data Management and Scoring

The collected questionnaires were checked for completeness. The data were coded, manually cleaned, and entered into Epi Info version 3.1 and exported to SPSS version 26. For data scoring, the correct answers were assigned a value of “1,” and incorrect answers or answers with “I don't know” were assigned a value of “0.” The correct answers for KAPs were separately summed, and their proportion out of the total for each category was calculated. The respondents were classified as knowledgeable, with positive attitudes and practice, when they had the score of 75% or above for each dependent variable (the KAP) [3].

### 2.6 Data Presentation and Analysis

Tables and figures were used for data presentation; data analysis was done using IBM‐SPSS version 26 software. The chi‐squared test was used to assess the level of association between categorical variables, and binary logistic regression analysis was conducted to identify factors associated with the dependent variables (the KAP). *p* < 0.05 was considered statistically significant at a 95% confidence interval.

### 2.7 Ethics and Consent

#### 2.7.1 Ethics Approval

Ethical clearance was obtained from the College of Natural and Computational Sciences Institutional Review Board (CNS‐IRB) on 31/08/2023, with reference number IRB/06/2015/2023. The investigation was done following the National Research Ethics Review Guideline, FDRE Ministry of Science and Technology, which is in accordance with international guidelines.

#### 2.7.2 Consent to Participate

After ethical clearance was obtained, a support letter was submitted to the school administration, and permission to conduct the study was obtained. For participants under 18 years of age, consent was obtained through the school administration, followed by assent from the students. For participants of age 18 years and above, written consent was obtained directly. Before the commencement of data collection, participants were informed about the purpose of the study, the procedure for data collection, the right to withdraw without negative consequences, anonymity, and confidentiality. Thus, the participation and all the data collected have been kept confidential and will be used only for the intended objectives.

## 3 Results

### 3.1 The Sociodemographic Characteristics of the Study Participants

A total of 377 students participated in the study with 100% response rate. The majority of the study participants were male (56.23%), 19 years of age (29.2%), Orthodox Christian (96.6%), Amhara ethnic group (100%), reside in the urban areas (66.8%), live alone (41.6%), are in ninth grade (30.2%), and are single (78.5%). The major source of information for the study participants about HIV/AIDS was radio/TV (42.7%). Most of the mothers of the students (27.9%) were unable to read. On the other hand, the majority of the fathers (35.2%) were educated up to high school level. Only 5% of the mothers had certificates and above compared to the fathers, whose proportion for the same category was 17%. Most of the family members had monthly incomes of more than 2000 Ethiopian Birr (ETB) (Table 1).

**TABLE 1:** Sociodemographic characteristics of the study participants.

Characteristics	Frequency (*n*)	Percentage (%)
Sex		
Male	212	56.23
Female	165	43.77
Age		
16 age	46	12.2
17 age	90	23.9
18 age	87	23.1
19 age	110	29.2
20 age	44	11.7
Religion		
Orthodox Christian	364	96.6
Muslim	13	3.4
Ethnicity		
Amhara	377	100
Residence		
Rural	125	33.2
Urban	252	66.8
Living status		
Parents	102	27.1
Alone	157	41.6
Friends	118	31.3
Education level		
Ninth	114	30.2
10th	101	26.8
11th	92	24.4
12th	70	18.6
Marital status		
Single	296	78.5
Married	81	21.5
Mother's education		
Unable to read	105	27.9
Can read and write	92	24.4
Grades 1–8	67	17.8
Grades 9–12	93	24.6
Certificate and above	20	5.3
Father's education		
Unable to read	55	14.6
Can read and write	52	13.8
Grades 1–8	73	19.4
Grades 9–12	133	35.2
Certificate and above	64	17
Family's monthly income (ETB)		
<500	69	18.3
(ETB)		
500–1500	85	22.5
1501–2000	92	24.4
>2000	115	30.5
Source of information		
Radio/TV	161	42.7
Literature/Poster	82	21.75
Friend	64	16.98
Family member	44	11.67
Health Institution	26	6.90

### 3.2 Knowledge About HIV/AIDS Transmission, Prevention, and Control (Good and Poor Knowledge)

**FIGURE 2:**
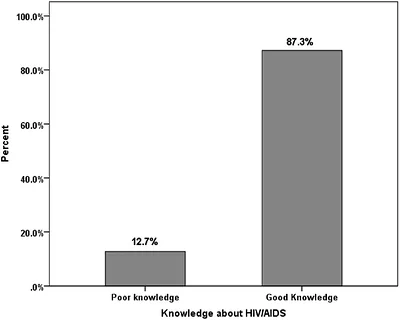
The level of knowledge of the students towards HIV/AIDS. HIV, human immune deficiency virus.

The majority of the study participants (87.3%) were knowledgeable (Figure 2), had positive attitude (88.9%), and had good practice (88.6%) towards HIV/AIDS. All of the students reported that HIV and AIDS are not the same, AIDS has no cure, HIV cannot be transmitted through saliva and sweat, taking shower immediately after sexual intercourse cannot prevent transmission of HIV, and sharing swimming pool with infected person cannot transmit HIV. The majority of the student had good knowledge about the causative agents, transmission, prevention, and control of HIV/AIDS. However, there were misconceptions as some students responded that correct and consistent use of condoms cannot prevent the transmission of HIV (23.3%), mosquitoes can transmit the virus (14.3%), being faithful to a single uninfected sexual partner cannot prevent HIV infection (6.9%), abstinence from sexual intercourse cannot prevent HIV (23.3%), an HIV‐infected person can be identified through physical appearances (15.4%), HIV can be transmitted through coughing and sneezing (22%), symptoms of HIV infection are visible within few days of infection (9.5%), there is a vaccine for HIV (21.5%), having multiple sexual partners is not a risk factor for contracting HIV (8.5%), and using one condom over the other better prevents HIV infection (20.2%) (Table 2).

**TABLE 2:** Distribution of respondents’ knowledge about human immunodeficiency virus (HIV)/AIDS.

Variables	Frequency (*n*)	Percentage (%)
Caused by bacteria		
Yes	26	6.9
No	297	78.8
I don't know	54	14.3
HIV and AIDS are same		
No	377	100.0
Consistent and correct usage of condoms prevents HIV		
Yes	289	76.7
No	88	23.3
AIDS has no cure		
Yes	377	100.0
Mosquito bite can transmit HIV		
Yes	54	14.3
No	323	85.7
HIV spreads through saliva or sweat		
No	377	100.0
Being faithful to a single sexual partner can prevent HIV		
Yes	348	92.3
No	26	6.9
I don't know	3	0.8
Abstinence from sexual intercourse can prevent HIV		
Yes	289	76.5
No	88	23.3
HIV‐infected person can be identified through physical appearance		
Yes	58	15.4
No	319	84.6
HIV cannot transmitted through coughing and sneezing		
Yes	294	78.0
No	83	22.0
Taking shower immediately after sexual intercourse prevents from being infected with HIV		
No	377	100.0
Symptoms of HIV infection are visible within few days of infection		
Yes	36	9.5
No	293	77.7
I don't know	48	12.7
All HIV‐infected mothers give birth to HIV‐infected children		
Yes	144	38.2
No	207	54.9
I don't know	26	6.9
There is vaccine for HIV		
Yes	81	21.5
No	296	78.5
Multiple sexual partners increase the risk of HIV infection		
Yes	345	91.5
No	32	8.5
Sharing swimming pool with infected person can transmit the virus		
No	377	100.0
Using condoms one over the other during sexual intercourse reduces the chance of HIV transmission		
Yes	76	20.2
No	234	62.1
I don't know	67	17.8

### 3.3 Attitude Towards HIV/AIDS

**FIGURE 3:**
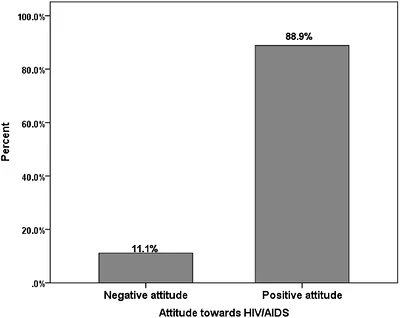
The level of attitude of the students towards HIV/AIDS. HIV, human immune deficiency virus.

The majority of the students (88.9%) had positive attitude towards HIV/AIDS (Figure 3). However, misconceptions and negative attitudes were also noted among the students regarding HIV and HIV patients. The misconceptions and negative attitudes include the problems caused by HIV is exaggerated (6.9%), only people who lead immoral lives are infected by HIV (34%), AIDS patients should be isolated for the safety of others (8.5%), traditional medicines can treat AIDS (4.2%), receiving services from HIV‐infected individuals have problems (11.9%), caring and support for HIV‐infected individuals have problems (44%), an individual infected by HIV/AIDS has got his/her price (37.4%), HIV positive individuals should not be given care when they are sick (6.9%), one should no more be a friend if the person is HIV positive (5.3%), items and goods should not be bought from an HIV positive shop keeper (4.5%), and an HIV positive student should not be allowed to continue her/his education (1.6%) (Table 3).

**TABLE 3:** Attitude towards human immune deficiency virus (HIV)/AIDS.

Variable		Frequency (*n*)	Percentage
Only those people who lead immoral lives will get HIV	Agree	128	34.0
	Disagree	249	66.0
AIDS patient should be isolated for the safety of others	Agree	32	8.5
	Disagree	345	91.5
AIDS can be treated by traditional medicines	Agree	16	4.2
	Disagree	361	95.8
The problems created by HIV/AIDS are exaggerated	Agree	26	6.9
	Disagree	351	93.1
Shaking hands with PLWHAs has risks	Disagree	377	100.0
Working with PLWHAs have no risks	Agree	377	100.0
Sharing room with PLWHAs will have risks	Disagree	377	100.0
Eating with PLWHAs have risks	Disagree	377	100.0
Sharing latrine with PLWHAs has risks	Disagree	377	100.0
Receiving services from PLWHAS has risks	Agree	45	11.9
	Disagree	332	88.1
Giving care and support for PLWHAs has risks	Agree	166	44.0
	Disagree	211	56.0
People who are infected with HIV through sexual intercourse have got their price	Agree	141	37.4
	Disagree	236	62.6
Give care for HIV positive relatives when they fill ill	Agree	351	93.1
	Disagree	26	6.9
Continue friendship after finding out HIV positive status	Agree	357	94.7
	Disagree	20	5.3
Buy item from an HIV‐positive shopkeeper	Agree	360	95.5
	Disagree	17	4.5
HIV‐positive student should be allowed to continue with her/his education	Agree	371	98.4
	Disagree	6	1.6

### 3.4 Practice Towards HIV/AIDS

**FIGURE 4:**
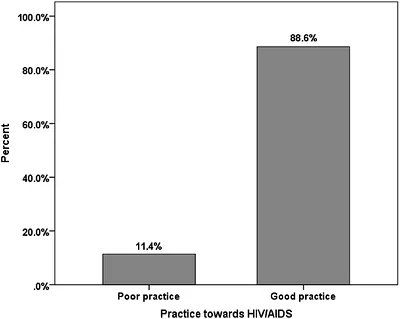
The level of the students’ practice towards HIV/AIDS. HIV, human immunodeficiency virus.

The majority of the students in the current study (88.6%) demonstrated good practice towards HIV/AIDS (Figure 4). A total of 49 (13%) individuals among the 377 students had sexual exposure; all of them used condoms during sex, and 9 of them were intoxicated during the sexual encounter. The majority of the students did not share sharp materials with their family (78.8%), though about 21.2% of the students reported sharing sharp materials with their family members. Most of the students (85.7%) were not tested for HIV. Among the students who had sexual exposure, the majority, 35 (71.43%) of them, were males.

### 3.5 Binary Logistic Regression Model

To fit the multivariate logistic regression model, independent variables with *p* ≤ 0.25 in the bivariate analysis were used. Sex, residence, grade level of the students, marital status, and living status were included as predictors of KAP after testing in the bivariate analysis. After adjusting for other variables in the model, only residence and grade level were the significant predictors of the knowledge of the students about HIV/AIDS. Students from urban areas were over six times more likely to be knowledgeable than those from rural areas (AOR = 6.52, CI: 2.22–19.12, *p* = 0.001). In addition, the odds of being knowledgeable increased as grade level increased from 9th to 12th, indicating that students from higher grades had greater odds of demonstrating knowledge compared to those from lower grades. Students in Grade 10 had 2.7 times higher odds (AOR, 2.70; CI: 1.15–6.36, *p* = 0.023), those in Grade 11 had 3.69 times higher odds (AOR, 3.69; CI: 1.47–9.30, *p* = 0.005), and those in Grade 12 had 8.55 times higher odds (AOR, 8.55; CI: 2.41–30.27; *p* = 0.001) of being knowledgeable than Grade 9 students.

Regarding the attitude towards HIV/AIDS, married students had three times more positive attitude towards HIV/AIDS than single individuals (AOR, 3.98, CI: 1.10–14.56, *p* = 0.037). The source of information about HIV/AIDS varied but the most preferred source of information was media (radio/TV), and students who obtained information about HIV/AIDS from their family had significantly lower positive attitude towards HIV/AIDS than those who obtained the information through media (radio/TV) (AOR, 0.30; CI: 0.11–0.80, *p* = 0.02). None of the independent variables predicted practice towards HIV/AIDS (*p* > 0.05). Unlike the knowledge, as grade level increased, the practice towards HIV/AIDS decreased. Similarly, married individuals who had better knowledge and positive attitude had lower practice towards HIV/AIDS. Students from urban areas had better practice than those from rural areas (Table 4).

**TABLE 4:** The knowledge, attitude, and practice of students towards human immunodeficiency virus (HIV)/AIDS.

Variables	Knowledge AOR (CI)	Attitude AOR (CI)	Practice AOR (CI)
Sex			
Male	1	1	1
Female	1.22 (0.82–1.83)	1.10 (0.55–2.22)	0.834 (0.43–1.61)
Residence			
Rural	1	1	1
Urban	6.52 (2.22–19.12)^***^	1.20 (0.57–2.51)	1.62 (0.82–3.23)
Grade level			
9th	1	1	1
10th	2.70 (1.15–6.36)^*^	1.0 (0.40–2.52)	0.74 (0.30–1.83)
11th	3.69 (1.47–9.30)^**^	0.80 (0.33–1.96)	0.60 (0.25–1.45)
12th	8.55 (2.41–30.27)^***^	1.20 (0.44–3.27)	0.77 (0.30–2.01)
Marital status			
Single	1	1	1
Married	2.15 (0.751–6.15)	3.98 (1.10–14.56)^*^	0.96 (0.42–2.23)
Living status			
Parents	1	1	1
Alone	2.30 (0.96–5.52)	1.62 (0.63–4.13)	1.25 (0.51–2.83)
Friends	1.13 (0.51–2.54)	0.84 (0.37–2.01)	0.88 (0.38–2.03)
Source of information			
Radio/TV	1	1	1
Literature	1.1 (0.49–2.76)	0.47 (0.19–1.16)	1.01 (0.43–2.38)
Friends	1.60 (0.57–4.48)	0.44 (0.15–1.28)	0.83 (0.32–2.13)
Family members	1.24 (0.44–3.45)	0.30 (0.11–0.80)^*^	0.59 (0.21–1.65)
Health institutions	1.26 (0.30–5.21)	0.98 (0.20–4.81)	0.81 (0.21–3.16)

*p ≤ 0.05.

**p ≤ 0.005.

***p ≤ 0.001.

### 3.6 The Association of Knowledge and Attitude With Practice

Knowledge about the transmission, prevention, and control, and attitude towards HIV/AIDS did not significantly differ from students’ practice towards HIV/AIDS. Though the differences were not statistically significant, being knowledgeable and having positive attitude was linked to safe practices towards HIV/AIDS (Table 5).

**TABLE 5:** The association between knowledge about and attitudes towards human immune deficiency virus (HIV)/AIDS.

Variables	Practice		*X* ^2^ (df = 1)	*p* value
	Unsafe practice	Safe practice		
Attitude				
Negative	7	35	1.295	0.255
Positive	36	299		
Knowledge				
Not knowledgeable	5	43	0.053	0.817
Knowledgeable	38	291		

## 4 Discussion

This study assessed the determinants of KAPs towards HIV/AIDS of high school and preparatory school students. Most of the study participants had good knowledge (87.3%), positive attitude (88.9%), and good practice (88.6%) towards HIV/AIDS. The level of knowledge was higher than reports from other parts of the country [13, 14, 15], as well as reports from other countries [16, 17, 18]. The majority of the respondents knew the causative agents, mode of transmission, prevention, and control of HIV/AIDS, which is in agreement with other reports in Ethiopia [8, 19, 20, 21, 22, 23], Ghana [17], South Africa [16], Tanzania [24], and Kuwait [25]. High levels of knowledge about the causative agents, transmission modes, and prevention methods of the disease could lead to safe sexual behaviors leading to reduced transmission of the disease. The positive attitude and good practice in the current study might suggest strong engagement of the school in HIV/AIDS education.

The respondents from higher education levels had significantly better knowledge than those from lower education levels. Similar associations were also reported from other parts of the country previously [6, 26] and from Lebanon [18], but different from studies in Ghana [17] and Kuwait [25]. The result of the study implies the importance of education in preventing HIV/AIDS and also the need for preparing simplified health education documents targeting lower grade students. The difference in the level of knowledge compared to reports from other countries might be due to cultural differences, data collection instruments, and prior information about the disease.

Respondents from urban areas had higher level of knowledge compared to those from rural areas, which was in agreement with a report from other parts of the country [6, 26]. This could be because respondents from urban areas had better access to information about HIV/AIDS through media devices, health centers, and better school settings. The differences in the knowledge level between rural and urban areas underscore the need for targeted intervention in rural areas.

The respondents in our study were well informed about HIV/AIDS and the main source of information was Radio/TV (Table 1). This result is in agreement with research done elsewhere in the country [8, 19, 21], but different from a report by Nubed and Akoachere [3], from Cameroon, where sex education in school took the largest part. This implies the need for context‐specific strategies to disseminate information about HIV/AIDS.

Though the majorities were well informed, there were misconceptions among fewer respondents regarding the causative agent of the disease, transmission, prevention, and control (Table 3). Misconceptions regarding the causative agent, transmission, prevention, and control are in agreement with previous reports: transmission of the virus through mosquito bite [3, 6, 8, 17, 21, 27], shaking hands [17, 19, 21, 25], sharing food or water [3, 19], coughing and sneezing [8, 15, 19, 21], and sharing the same bathroom [8, 17, 19]. AIDS is caused by bacteria or other causative agents and has no cure, and vaccines are similar to reports in different parts of Africa [3, 8, 17, 19, 23, 24, 28]. Similar to our report, respondents in a study by Nubed and Akoachere [3] thought that healthy‐looking individuals cannot have HIV/AIDS [3, 6]. The overlap in the misconception among respondents from the same country as well as different areas of the world depicts the importance of trainings directly focused on addressing common misinformation about HIV/AIDS in schools and communities.

The majority of the study participants had positive attitudes towards people living with HIV/AIDS, though smaller proportion of the respondents had negative attitudes (Table 3). The proportion of individuals with negative attitudes (34%) reported by Shiferaw et al. [8] is much higher than the value (11.1%) in our report. Some of the negative attitudes similar to our report include as follows: the association of the disease with immoral life style [8, 20], isolation of people living with HIV/AIDS [8, 17], HIV positive student should not continue his/her education [17, 19], social life with HIV positive individuals should be avoided [17, 19, 21], cannot care for HIV positive individuals during illness [3, 17, 19], quit friendship after knowing HIV positive status [3, 17, 19], and cannot purchase item from HIV positive individuals [3, 17, 19, 25]. The persistence of negative perception shows that stigma is deep‐rooted due to lack for appropriate information. This shows the need of continued anti‐stigma campaigns.

From the multivariate analysis, married respondents had about four times positive attitude towards HIV/AIDS‐positive individuals than those who were single. But respondents who obtained information from their family had negative attitudes (Table 3). Like our study, higher level of positive attitude was reported for married individuals [29]. However, investigators from Ghana [17] reported more positive attitude among single individuals than the married ones. The differences in the attitudes of respondents could be attributed to differences in their cultural characteristics and the level of prior awareness about the disease prior to collecting the data.

The overall good practice of the respondents in the current study was rated good (88.6%) (Figure 3). But none of the independent variable significantly were associated with practice towards HIV/AIDS (Table 3). The overall good practice is higher than previous reports by other investigators [13, 14, 15, 17, 30]. Compared to our study, lower level of condom uses were reported from different parts of the country [30, 31, 32] and other countries [18, 33, 34] and 10 different African countries [35]. Having sex while being intoxicated or under the influence of drugs was also reported by investigators from Ethiopia [8, 30], Tanzania [33], Kenya [34], Cameroon [3], Ghana [17], and 10 African countries [35]. Having sex with multiple sexual partners was also reported by other investigators [21, 30, 35]. In accordance with our report, a study by Nubed and Akoachere [3] reported that healthy looking person cannot have HIV infection, abstaining from sex cannot reduce the risk of HIV, having sex with only one faithful uninfected partner cannot reduce HIV transmission were among the misconceptions in sexual practices that could lead to contracting HIV/AIDS. Significant associations were not observed between the knowledge and attitude level with practice of the respondents towards HIV/AIDS (Table 6). Similarly, Semunigus et al. [19] also reported the absence of significant association between the level of knowledge about HIV/AIDS and HIV/AIDS‐related practices. In other investigations, knowledge of HIV/AIDS was not significantly associated with attitude towards HIV/AIDS [7, 18]. In our study, all of the study participants who had sexual exposure used condoms; however, some of them reported having sex under the influence of drugs or intoxication, which could lead to contracting the disease due to improper use of condoms or aberrations of the condom during sexual activity. The lack of correlation between attitude and practice implies that behavioral changes might be affected by external factors like access to resources and peer influence. In a country like Ethiopia with over 80 different ethnic groups, the differences in the level of practices could also be attributed to differences in the cultural and religious backgrounds. In addition, factors such as sex education and access to different information outlets like internet could affect the level of practices. Thus, strong evidence‐based awareness creation campaigns and targeted intervention to address misinformation about HIV/AIDS transmission, prevention, and causative agent, and actively working to reduce stigma against people living with HIV/AIDS are crucial.

**TABLE 6:** Practices of the students towards human immunodeficiency virus (HIV)/AIDS.

Variables	Frequency (*n*)	Percentage
Have sexual exposure		
Yes	49	13.0
No	328	87
Used condom during sex		
Yes	49	100
Intoxicated during your last sexual encounter		
Yes	9	18.4
No	40	81.6
Share sharp materials with family members or friends		
Yes	80	21.2
No	297	78.8
Tested for HIV in the last three months		
Yes	54	14.3
No	323	85.7

## 5 Conclusions

The level of KAP in the current study about the causative agents, transmission, prevention, and control was rated good (>75%). However, there were prevailing misconceptions among few respondents regarding the causative agents, transmission, prevention, and control of the disease. Similarly, lower proportion of the respondents had negative attitudes towards people living with HIV/AIDS, which could potentially lead to stigma and discrimination affecting the international as well as national targets for the control of the disease. Sex education in schools is inevitable to avoid misconceptions about the disease to increase the level of safe practices and stigma against HIV/AIDS‐positive individuals.

## 6 Limitation of the Study

This study was done in a preparatory and high school in the northern part of Ethiopia. Thus, it cannot be generalized to other regions of the country due to differences in culture and socio‐economic features. Similarly, despite being told the study was anonymous, there is still bias in openly reporting issues related to HIV/AIDS and past sexual behaviors that could affect the result. In addition, the use of cross‐sectional design, which provides only a snapshot of the disease despite the dynamic nature of the HIV epidemic, is also another limitation. The purposive sampling technique used for selection of the study setting can also limit the generalizability of the study. Despite this, the study has contributed in assessing the level of awareness about the disease, misconceptions, and practices among high school students, which could be used by other researchers as a baseline in the study area for further investigation and intervention. The association between various factors could also help to guide future research and guided interventions.

## Author Contributions

**Tilahun Adugna**: conceptualization, methodology, software, data curation, investigation, validation, formal analysis, visualization. **Solomon Gezahegn**: conceptualization, methodology, software, data curation, formal analysis, writing – and reviewing. **Mujib Abdulkadir**: conceptualization, methodology, writing – review and editing. **Fitsum Tigu**: conceptualization, methodology, writing – review and editing. **Asnake Desalegn**: conceptualization, methodology, software, data curation, validation, formal analysis, visualization, supervision, writing – original draft, review and editing.

## Funding

The authors have nothing to report.

## Conflicts of Interest

The authors declare no conflicts of interest.

## Data Availability

The data that support the findings of this study are available from the corresponding author upon reasonable request.
